# Pregnancy-Associated Osteoporosis Presented with Femoral Neck Fracture: A Case Report and Literature Review

**DOI:** 10.31661/gmj.v9i0.1750

**Published:** 2020-12-26

**Authors:** Azam Faraji, Zahra Shomali, Sedigeh Yoosefi

**Affiliations:** ^1^Maternal-Fetal Medicine Research Center, Shiraz University of Medical Sciences, Shiraz, Iran; ^2^Obstetrics and gynaecology Department, Medical School, Shiraz University of Medical Sciences, Shiraz, Iran; ^3^Maternal-Fetal Medicine Research Center, Perinatology Department, Shiraz University of Medical Sciences, Shiraz, Iran

**Keywords:** Osteoporosis, Pregnancy, Fracture, Femur

## Abstract

**Background:**

Pregnancy-associated osteoporosis (PAO) is a rare condition characterized by reduced bone mineral density during the third trimester or lactation. Multiple risk factors, such as genetic, sedentariness, and 25-hydroxy vitamin D deficiency, are associated with PAO. In the majority of cases, PAO is presented with a compression fracture in vertebras, but in our case, it presented with a fracture of the femoral neck. **Case Presentation:** A 29-year-old, gravida one woman, developed right hip joint pain during the gestational age 34 weeks who referred to our clinic. Despite conservative management, the pain intensified. The patient delivered a healthy neonate in the 38th week of gestation and afterward underwent magnetic resonance imaging of the pelvic, revealing a bruised bone in the femoral neck. Since she had developed a femoral neck fracture during the postpartum period, she underwent open reduction and fixation of the femoral neck. Dual-energy X-ray absorptiometry (DEXA) revealed osteoporosis of the vertebras and femoral neck. She received calcium supplements and alendronate, and the pain was relieved. On 2-year and 4-year follow-up, she was found to be osteopenic with significant improvement in DEXA indices.

**Conclusion:**

PAO is a rare condition among young women. This condition should be kept in mind when hip joint or back pain is encountered during pregnancy.

## Introduction

Pregnancy and lactation are conditions that cause negative calcium (Ca) balance –due to fetus demand– that can be improved by the absorption of Ca through the maternal skeletal system [1]. Pregnancy-associated osteoporosis (PAO) is a rare condition with an incidence of 0.4/100,000 [2]. Its signs and symptoms (i.e., low back and pelvic pain) are common in pregnancy, so everyone that works in the obstetric field should understand it. At first, Nordin and Roper described PAO in 1955 by presenting four cases with confirmed PAO with bone biopsy [3]. PAO can lead to multiple compression fractures, leading to morbidity in mothers, such as shortening of height and inability to walk [4].

## Case Report

**Figure 1:**
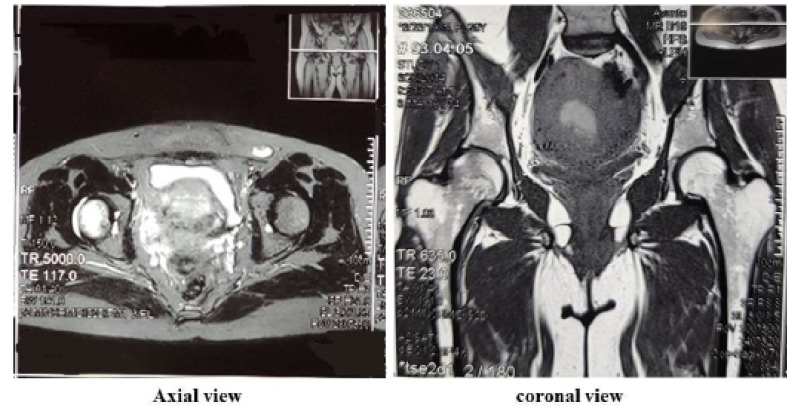
Magnetic resonance imaging (axial and coronal view) revealed bone marrow edema in the right femoral head

**Figure 2:**
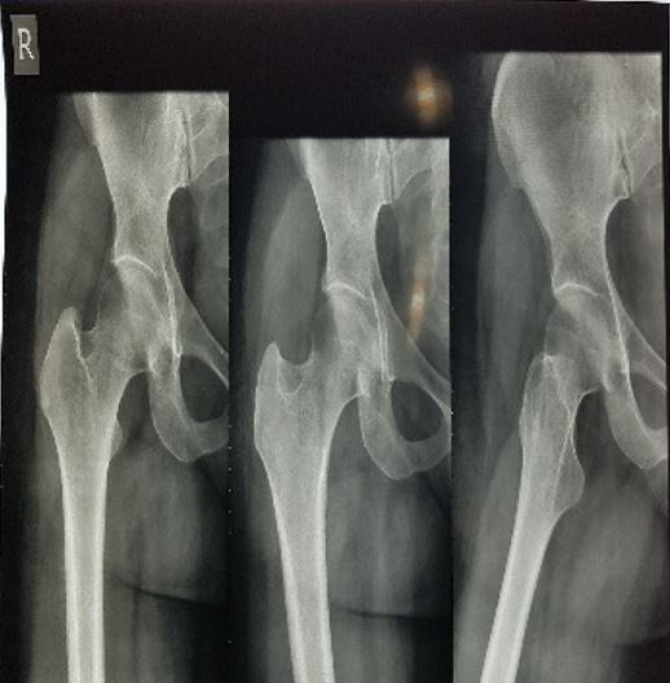
Pelvic X-Ray in favor of femoral neck fracture

**Figure 3:**
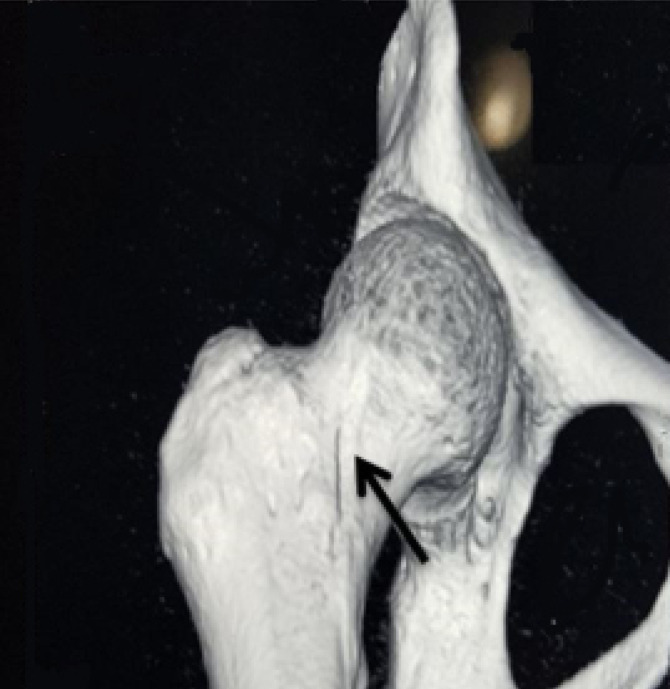
Spiral CT scan that shows the possibility of a small femoral neck fracture (arrow)

 A 29-year-old gravid one woman with body mass index 22 (before pregnancy) referred to our outpatient clinic in 34 weeks of gestation with acute pain in her right hip joint since ten days ago. There was no any past medical and social history. The pain was associated with limping, which had progressively increased during the past ten days. It was alleviated by rest and exacerbated on ambulation. The physical examination of the right hip joint revealed a reduced range of motion of 0–90° for flexion and 20° for external rotation, limited by pain, whereas rotation of the left hip showed no limitation. She had an orthopedic visit administrating analgesics without any improvement. Therapy included unloading the joint with crutches and exercise. Hence, hip magnetic resonance imaging (MRI) was requested, which was refused by the patient. She was admitted to our center due to fetal distress and diagnosed with the abnormal non-stress test during the 38th week of gestation. She delivered through cesarean section and gave birth to a healthy female neonate with an APGAR score of 10 and birth weight of 3150g. After the delivery, patient hip joint pain increased significantly; hence, a pelvic MRI was performed revealing increased intensity in Short Tau Inversion Recovery (STIR) T2-weighted and matching decreased intensity in T1-weighted images of the right femoral head without a collapse in favor of bone bruising extending to the femoral neck (Figure-1). The left femoral head and neck were found to be normal. She was diagnosed with transient osteoporosis of the hip; thus, Ca-D and analgesics were prescribed in therapeutic dosage. Her pain aggravated during the following week, and she was unable to walk with severe limping. Pelvic radiography was performed, revealing the collapse of the right femoral neck in favor of femoral neck fracture (Figure-2). The spiral computed tomography (CT)-scan of the right hip joint was done to confirm the diagnosis that showed small stress fracture of the femoral neck was suspicious (Figure-3), due to patient’s pain and the decision of orthopedic team open reduction and cannulated screw fixation of right femoral neck fracture was performed. Postoperatively, a rheumatologist was consulted, who requested dual-energy X-ray absorptiometry (DEXA) of the lumbar vertebras 2-4 showing a Z-score of –2.7 and a bone mineral density (BMD) of 0.877 g/cm2, representing osteoporosis. The DEXA of both femoral necks revealed osteoporosis (Z-score=-2.3). For finding the cause of osteoporosis in this patient, Ca, phosphorus, and parathormone (PTH) serum levels were checked that were in the normal range. Also, all the other laboratory data, including 24-hour urine for Ca secretion and liver and thyroid function tests showed no any abnormal finding (Table-1). Hence, hyperparathyroidism, hyperthyroidism, renal dysfunction, excess Ca excretion, and liver disease were excluded. Only 25-OH-vitamin D serum level was found to be insufficient. A total of 1000 units of daily Ca supplements and vitamin D along with 70mg weekly alendronate were prescribed for the patient. The patient advised to the cessation of breastfeeding. After a month, the pain was relieved and she was able to walk without limping. On a 2-year follow-up, the DEXA of lumbar vertebrae 2-4 (Z-score=-1.4) and hip revealed (Z-score=-2.2) a significant improvement in bone mineral density, in 4-year follow-up Z-score in lumbar vertebras and hip were -1.5. At present, she takes part in her daily routine activities without any concern, but she is still under observation.

## Discussion

The most common clinical symptom of PAO is back pain during late pregnancy or lactation, and related symptoms are hip pain (unilateral or bilateral), difficulty in weight-bearing until nearly immobilized [1]. PAO etiology is unclear, but multiple hypotheses regarding this condition are in the literature; such as genetic factors (mutation in some genes such as *LRP5*, *COL1A1*, and *COL1A2*) [4], increased in PTH-rp secretion by breast and placenta that might lead to bone absorption during pregnancy and lactation [5,6] and increase Ca demand for fetus skeletal system [1,7-9]. Vit D deficiency is common in these patients [8]. Other risk factors include low BMI, sedentariness, poor nutrition (vegetarians) [7-9], smoking, and low Ca intake. Aproximallty 82% of all cases have at least one of these risk factors [8]. To diagnose PAO, the exclusion of secondary causes is vital [9,10]. These causes include hyperparathyroidism, hyperthyroidism, drugs (e.g., heparin and corticosteroid), chronic kidney disease, liver disease, and cancer [10]. In our case, the patient had no past medical history, and she was not a smoker and/or vegan. The laboratory findings were all normal, as mentioned before, except for the 25-OH Vit D serum level, which was insufficient. She has not a history of use of any drug, especially corticosteroid and heparin during pregnancy or before it. All in all, the idiopathic nature of this disease makes it challenging to predict it during pregnancy. Its diagnosis should be based on the assessments that are done for prolonged pain or fragility fractures. X-ray and MRI might be performed, but pregnant women might refuse it (such as our case). X-ray shows decrease bone trabeculation and cortical thinning [11] or a sing of fragility fractures. In our case, MRI was done to evaluate hip pain, which revealed bone marrow edema, hypointense T1-weighted signals, and hyperintense T2-weighted signals, these findings are seen in transient osteoporosis of hip [12]. DEXA confirms the diagnosis, which must be done for patients with fragility fractures during the premenopausal period [10]. Interpretation of DEXA in premenopausal patients is different from post-menopausal, and World Health Organization classification –that is used for post-menopausal patients– should not be used for these patients; Z-score and not T-score should be used for the interpretation of DEXA (Z-score bellow -2 is used to confirm the osteoporosis) [13]. As therapeutic measures, supplementation by Ca and vitamin D and the use of bisphosphonates are recommended [7-9]. Bisphosphonates are in C category for safety in pregnancy and lactation due to abnormal findings in pregnant rats. Bisphosphonates have long term bounding to the bone, and risk of fetal exposure in future pregnancies should be kept in mind; however, in the literature, no anomaly was observed in fetuses with unwanted exposure to these drugs. Thus, bisphosphonates in this group of patients should be used case by case [10]. Other medications such as calcitonin are used, but there are no many experiences about them in the literature. The cessation of breastfeeding is advised [8,9]. In some cases, orthopedic procedures such as open reduction of fractures, bracing or physical inactivity are warranted. Mothers and their babies are at risk of long term osteoporosis, and this condition might not resolve for years [1,8]; hence, long term follow-up is recommended. DEXA should be repeated during therapy to evaluate the response to medication. PAO is a rare condition and should be kept in mind when a pregnant or postpartum woman complains of hip or back pain that does not respond to usual medications. To conclude, these patients demand close and long term follow-up to prevent complications. In the meantime, we have to look for secondary causes of osteoporosis in premenopausal women.

## Acknowledgment

 We would like to thank the patient and her family for good cooperation. The authors wish to thank Mr. H. Argasi at the Research Consultation Center (RCC) of Shiraz University of Medical Sciences for his invaluable assistance in editing this manuscript.

## Conflict of Interest

 The authors declare that they have no conflict of interest.

**Table 1:** Laboratory Findings Associated with Osteoporosis

**Laboratory findings **	**At diagnosis**	**2-year**	**4-year**
**WBC**	6800	5700	6000
**Hb (gr/dl/HCT)**	12.9/38.9%	13.5/40.7%	12/37%
**Platelet count **	385000	256000	235000
**ESR**	9	7	4
**CRP**	Negative	Negative	Negative
**Vit D (ng/ml)**	6.03	25	30
**TSH ( mIU /L)**	1.92	1.4	0.95
**Paratormone ( pg /ml)**	50	63	
**BUN/Creatinine **			20/0.8
**Calcium (mg/dl)**	9.1	9.3	9.2
**Phosphorous (mg/dl)**	3.2	3.2	3.9
**Alkaline phosphatase (IU/L)**	187	131	99
**SGOT/SGPT**			15/15
**Tissue transglutaminase** **IgG-IgA AU/ml**	4.3-<0.3		
**24-hour urine ** **volume/ calcium / creatinine **	950ml/82mg/751		
**Anti-endomysium IgA**		Negative	

**WBC: **White blood cell; **Hb:** Hemoglobin; **ESR**: Erythrocyte sedimentation rate; **CRP: **C-reaction protein; **TSH: **Thyroid stimulation hormone; **BUN**: Blood urea nitrogen
